# Effects of Conformational Sampling on Computing Redox Properties Using Linear Response Approach

**DOI:** 10.1021/acs.jpcb.5c01121

**Published:** 2025-07-14

**Authors:** Suman Maity, Ronit Sarangi, Atanu Acharya

**Affiliations:** † Department of Chemistry, 2029 Syracuse University, Syracuse, New York 13244, United States; ‡ BioInspired Syracuse, Syracuse University, Syracuse, New York 13244, United States

## Abstract

Redox processes are an important step in many chemical and biochemical reactions. One simple approach to calculate the free energy change of a redox process is linear response approximation (LRA). However, variability in conformational and energy-gap sampling poses a challenge in balancing computational cost and accuracy. Herein, we calculate the redox properties of the one-electron oxidation processes for small, biologically relevant redox-active molecules (e.g., phenol, phenolate, benzene, indole, lumiflavin) in aqueous solution using two conformational sampling strategies. We sampled the conformations using molecular mechanics (MM) and hybrid quantum mechanics/molecular mechanics (QM/MM) simulations to investigate how these techniques affect redox properties. We also performed QM/MM energy-gap sampling while varying the QM region to investigate its impact on overall redox behavior. We observed free energy of oxidation, and consequently, oxidation potential differs consistently by ∼0.2–0.4 V between QM/MM and MM sampling for the molecules under investigation. Overall, we infer that computationally cheaper MM sampling would be adequate for computing the redox properties of small molecules when corrected by a system-specific correction factor.

## Graphical abstract

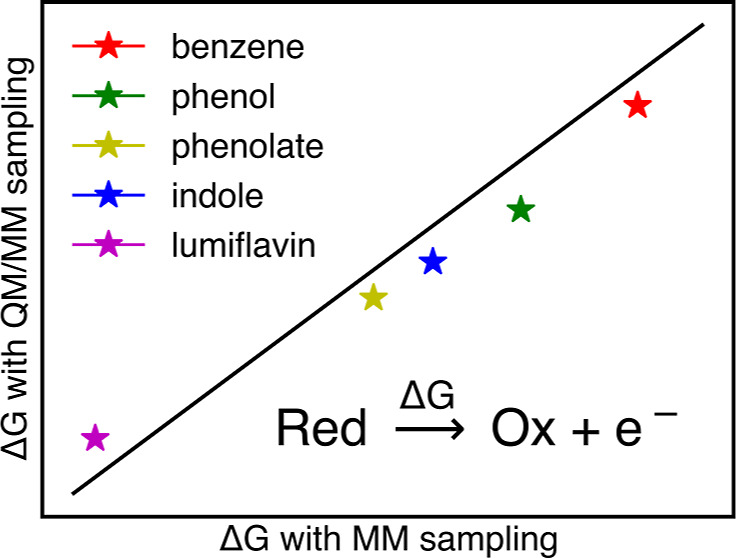

## Introduction

Redox reactions are fundamental to numerous electrochemical applications and essential biochemical processes, such as photosynthesis,^1^ respiration,^2^ metabolism,^3^ and cellular signaling.^4,5^ These reactions are governed by their free energy change (Δ*G*°) and redox potential (*E*°), which determines the ease with which a molecule can accept or donate electrons. Computation of redox properties provides detailed insights at a molecular level, particularly in cases involving multiple redox centers. However, the accuracy of such calculations depends on the appropriate sampling and modeling of the environment around the redox-active molecule. The explicit solvation model accurately describes short-range solute–solvent interactions, albeit at a higher computational cost. Note that the accuracy also depends on the size of the solvation shell, with larger solvent regions offering better alignment with experimental results.^6,7^

Among the widely used methods for free energy calculations, thermodynamic integration (TI)^8^ and free energy perturbation (FEP)^9^ require extensive sampling of the intermediate states connecting the reduced and oxidized states. Linear response approximation (LRA)^10,11^ provides a cost-effective alternative approach. Using LRA, the Δ*G*° of the redox reaction can be calculated by sampling only the end states, i.e., reduced and oxidized states.^12,13^ In this context, a common practice is to generate conformational sampling followed by single-point energy calculations to sample the vertical energy gaps (VEGs) using a ΔSCF approach.^14–16^

Conformational sampling can be performed using classical molecular dynamics simulations (MM), *ab initio* molecular dynamics (AIMD) simulations, and hybrid quantum mechanics/molecular mechanics (QM/MM) simulations. Although AIMD provides a robust description of the solute–solvent interaction, it is computationally demanding and can only be applied to small systems.^17^ The QM/MM simulation is often used for larger systems, where chemically relevant part of the system is treated quantum mechanically, and the rest is treated with a classical force field. However, the QM/MM sampling is still computationally very expensive, depending on the size of the QM region. Therefore, conformational sampling is often performed at a molecular mechanics level. Note that regardless of which potential guides the motion of a molecule, enhanced sampling techniques are typically essential to surmount high free-energy barriers.^18–21^ As expected, computed properties, such as protein–ligand binding affinity,^22^ energy levels of the DNA nucleobases,^23^ absorption properties^24^ are often sensitive to how the conformational sampling is generated. While alternative approaches, such as MM simulations followed by local optimization, behave reasonably well for structural parameters,^25^ they may not be effective for electronic properties.^26^ The relationship between redox properties and the sampling approach, particularly for biologically relevant molecules, has not been thoroughly examined in the current literature.

In this study, we investigate the effects of conformational sampling on redox properties of a selected set of systems. We use both MM and density functional theory (DFT) based hybrid QM/MM methods to sample conformations of oxidized and reduced states of our systems. Then, we use the QM/MM single-point calculations to estimate the VEGs for all conformations, followed by free energy calculations using the LRA approach. For this investigation, we focus on biologically relevant redox-active molecules: benzene, phenol, phenolate, indole, and lumiflavin (Figure 1). These molecules can participate in biological redox processes since they are aromatic in nature. For example, phenol and phenolate are involved in class I ribonucleotide reductases,^27^ photosystem II,^28–30^ cytochrome *c* oxidase,^31–33^ green fluorescence protein.^34^ Indole (side chain of tryptophan) is involved in metabolism^35^ and sleep cycle.^36^ Lumiflavin is the redox center of the flavin group^37,38^ which is involved in several enzymatic processes.^39–42^ These molecules have also been proposed as charge carriers in long-range charge transport.^43,44^ Therefore, it is very important to assess the impact of sampling protocol on the redox properties of these molecules, especially since AIMD and QM/MM techniques are out of reach for most biologically relevant systems.

**Figure 1:**
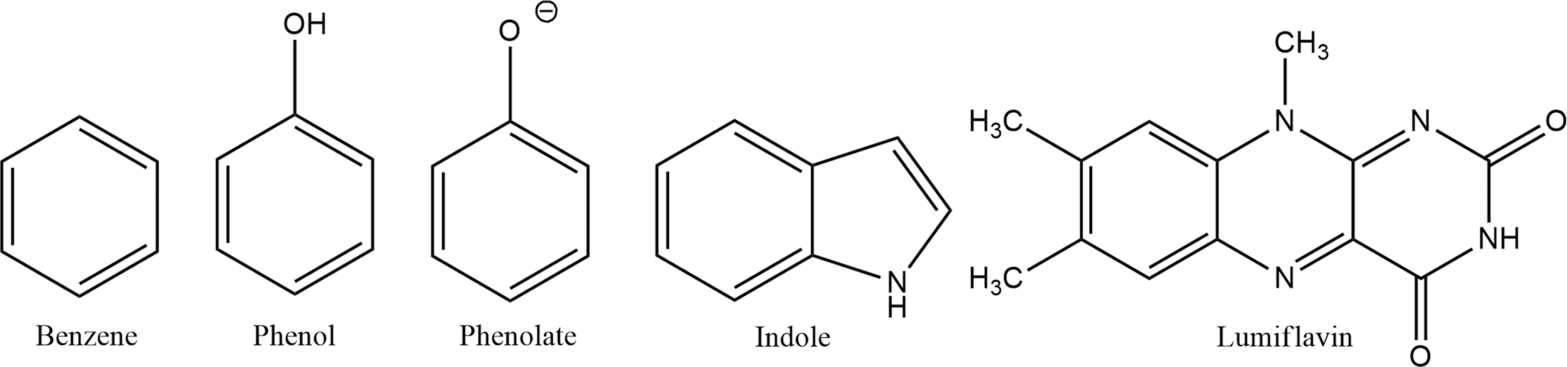
Systems used in this study. Benzene, phenol, phenolate, and indole are shown in their reduced form, while lumiflavin is shown in its oxidized state. A pictorial representation of one-electron oxidation process is shown in Figure S1.

## Methods

### Background

The one-electron oxidation processes can be represented by the following reaction.

$$Reduced\to Oxidized+e^{-}$$

The free energy change of this reaction is determined using the energy gap (Δ*E* or VEG) between final and initial states as the reaction coordinate.^9,45,46^

1 $$\Delta G_{ox}^{{}^{\circ}}=-k_{B}T\;\ln\langle \exp{\left[-\Delta E/k_{B}T\right]\rangle }_{i}$$

where ⟨···⟩_*i*_ denotes ensemble average over state *i*, *k*_B_ is the Boltzmann constant, and *T* is the temperature. According to the LRA approach, eq 1 is truncated after the first term, i.e., $G_{ox}^{{}^{\circ}}$ = ⟨Δ*E*⟩_red_ and Δ $G_{ox}^{{}^{\circ}}$ = ⟨Δ*E*⟩_ox_ for the reduced and oxidized states, respectively. Note that Δ*E* and VEG are used interchangeably throughout this article. Combining these expressions, we calculate the free energy change of one-electron oxidation processes as follows, (Figure 2).

2 ΔGox°=12⁡[⟨Eox−Ered⟩red+⟨Eox−Ered⟩ox]=12⁡[⟨ΔE⟩red+⟨ΔE⟩ox]

**Figure 2:**
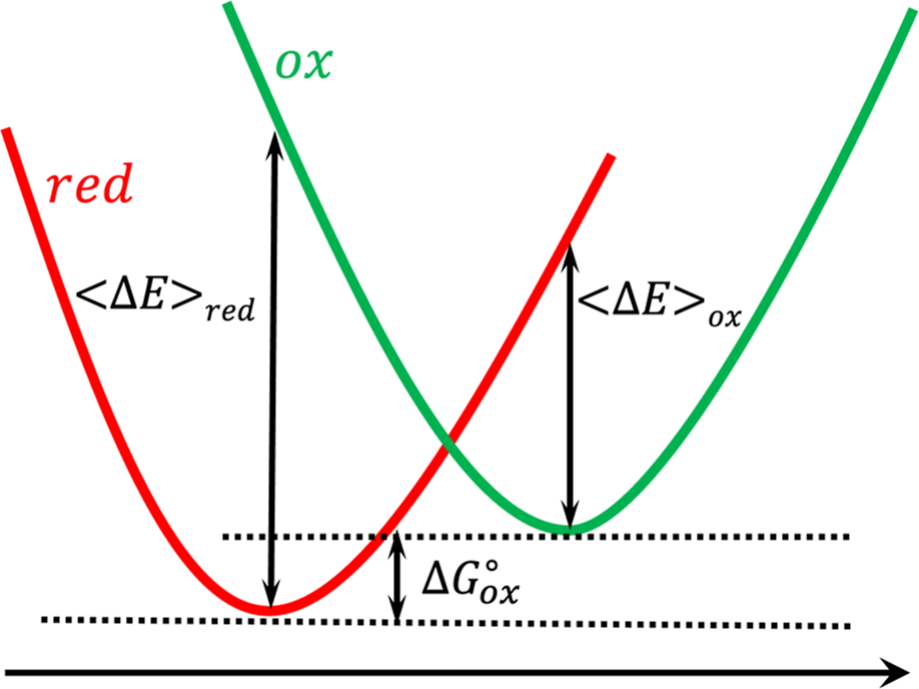
Free energy surfaces for an oxidation process where ⟨Δ*E*⟩_red_ and ⟨Δ*E*⟩_ox_ represent ensemble average VEGs for the reduced (red) and oxidized (ox) conformations, respectively. Δ $G_{ox}^{{}^{\circ}}$ is the free energy change of the oxidation process.

The oxidation potential (*E*°) relative to the standard hydrogen electrode (SHE) is computed using the Nernst equation

$$E^{{}^{\circ}}=\frac{{\Delta G}_{ox}^{{}^{\circ}}-{\Delta G}_{SHE}^{{}^{\circ}}}{nF}$$ 3

where *n* is the number of electrons involved in the redox process, *F* is the Faraday constant, and Δ $G_{\mathrm{SHE}}^{{}^{\circ}}$ is the free energy change for the oxidation of SHE.

### Computational Protocol

To investigate the impact of conformational sampling on redox free energy and oxidation potential, we performed two sets of simulations to sample the oxidized and reduced conformations of our systems. Following these simulations, we performed single-point hybrid QM/MM calculations to obtain the VEGs for calculating Δ $G_{ox}^{{}^{\circ}}$ (eq 2). The details of these calculations are described in the following sections and a workflow schematic summarizing the protocol is presented in Figure S2 of the Supporting Information.

### Force Field Parameters

The initial force field parameters for the reduced ground state (oxidized state for lumiflavin) were taken from the CHARMM general force field (CGenFF).^47^ Then, we have modified the parameters for both the oxidized and reduced states similar to a protocol adopted by Bravaya and co-workers.^6^ First, geometries of the oxidized and reduced states of the system were optimized in the gas phase at ωB97XD/6-31+G* level of theory using Gaussian 16.^48^ Then, we performed a natural bond orbital (NBO) analysis in the optimized geometry of the molecules using the same level of theory. In the modified force field, we used NBO charges as the partial charge and equilibrium bond length and bond angle values from the optimized geometry. All the other parameters such as force constants and the rest of the nonbonded parameters were taken from CGenFF, and they remained the same for both oxidation states. This approach was used in previous study of similar molecules.^6^

### Conformational Sampling

The conformations of the systems were sampled using classical molecular dynamics (MM simulation), and DFT based hybrid QM/MM simulations. In the QM/MM simulations, the solute (system of interest) was treated quantum mechanically with an electrostatic embedding scheme, where the surrounding solvent was described by the TIP3P water model.^49^ The force field parameters for both states of the systems were obtained as described in the previous section. The system was prepared in a 50 × 50 × 50 Å^3^ cubic box with the solute in the center of the box and a counterion placed randomly for charge neutrality, when necessary. The size of the water box was selected to account for important long-range solvation effects on VEGs, as reported in ref 6.

For MM simulations, we first ran a 2 ns constrained simulation to equilibrate the solvent density, keeping the solute molecule fixed. This was followed by a 1 ns NPT equilibration simulation where everything was allowed to relax. Then a 2 ns NPT production simulation was performed with 1 fs time step. We extracted 500 frames from the production simulation for single-point QM/MM energy calculations. All simulations were performed at 310 K temperature (with Langevin thermostat) and 1 atm pressure (with Langevin piston barostat^50,51^ ) using periodic boundary conditions (PBC). Short-range and long-range nonbonded interactions were evaluated every 1 and 2 steps, respectively. Long-range electrostatic interactions were calculated using the particle-Mesh Ewald (PME).^52^ In addition, we used a cutoff distance of 12 Å for nonbonded interactions and a switching function was used from 10 to 12 Å. The SHAKE and RATTLE algorithms were used to set the water O–H bonds rigid.^53^ These are common practices used to expedite molecular dynamics (MD) simulations for large macromolecular systems.

In the QM/MM conformation sampling, the solute in the QM region was treated at the BP86/6-31+G* level of theory, while the solvent and any ions were described classically. The BP86 functional was previously used to capture solute–solvent interaction in similar DFT/MM simulations.^54^ The integration time step for the QM/MM simulations was set at 0.5 fs. The full electrostatic potential was evaluated every step since the QM charges were updated every step. All the other simulation parameters were the same as the MM simulation. The equilibrated system from the previous MM simulation was used to perform a 5 ps NPT QM/MM equilibration simulation, where everything was allowed to relax. This was followed by 10 replicas of 5 ps QM/MM production simulations (total 50 ps) with a 0.5 fs time step. For each system, 500 frames were saved at 100 fs intervals for single-point QM/MM energy calculations. The MM and QM/MM simulations were performed using NAMD^55,56^ and ORCA/NAMD interface,^57^ respectively.

### Single-Point Energy Calculations

A hybrid QM/MM scheme was used for the single-point calculations, where the QM region was treated at the ωB97MV^58^/6-31+G* level of theory, while the MM region was treated using electrostatic embedding with point charges. The ωB97MV functional was chosen since it is a long-range corrected hybrid density functional and was reported to provide the lowest error (∼0.11 eV) for ionization energy across a wide range of molecules.^58^ Our benchmark calculations (Table S1) and others^15^ have also shown that the double-ζ basis sets with diffuse function on the heavier atoms are necessary for VEG calculations. We also examined the dependence of VEG on the size of the QM region using conformations obtained from QM/MM simulations. A QM cutoff radius was defined from the center of mass of the redox-active molecule for the construction of the QM region of different sizes. The “QM cutoff 0.0” is defined as only the solute molecule in the QM region. In contrast, the solute and all water within the cutoff radii are included in the QM region of the QM/MM single-point calculations for other cutoffs. The QM cutoff radii were varied from 4.0 to 7.5 Å with 0.5 Å increments to check convergence of VEG (Figure 3a). The average number of water molecules in the QM region for different QM cutoffs is reported in Tables S2. An alternative selection criterion based on shortest distance was tested for a smaller subset (Table S3). All QM/MM single-point energy calculations were performed using Q-Chem 6.0.^59^

**Figure 3:**
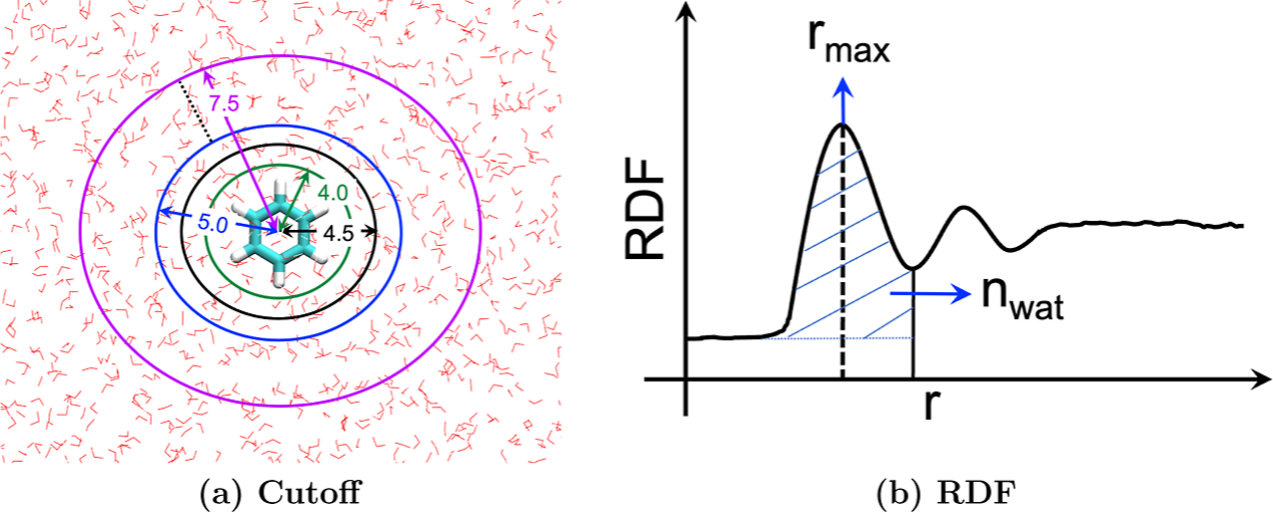
Cartoon figure of (a) cutoff analysis with different sizes of the QM region for single-point energy calculations and (b) radius of the first solvation shell (*r*_max_) and number of water molecules (*n*_wat_) within the first solvation shell in radial distribution function (RDF).

## Results and Discussions

In this section, we will examine the differences between force field based MM and hybrid QM/MM sampling protocols using structural parameters such as radial distribution function (RDF), radius of the first solvation shell, and number of water molecules within this shell. Additionally, we explore the effects of these conformational sampling protocols on VEGs and the influence of the QM region size in single-point QM/MM calculations. We also explore the effect of solute-counterion distance on VEGs. Lastly, we present the calculation of Δ $G_{ox}^{{}^{\circ}}$ and *E*° and compare them with the available experimental results.

### Solvation Behavior Changes Depending on Conformational Sampling Approach

The RDF analysis provides structural insights into the nearest-neighbor coordination and captures short- and long-range information about solvent distribution. The radius of the first solvation shell (*r*_max_) and the number of water molecules within this shell (*n*_wat_) can be determined from the position of the first maxima and the area under the first peak of the RDF, respectively (Figure 3b). However, due to the complexity of the RDFs (Figure S3), the estimation of *n*_wat_ is not trivial and can be ambiguous. Therefore, we compare RDF plots and *r*_max_ from MM and QM/MM simulations to analyze the variations in the local solvent structure around the solute. Figure S3 shows the RDF between the heavy atoms of the solute and the oxygen of water. The *r*_max_ values estimated from the RDFs for both the oxidized and the reduced states are shown in Table 1. Note that due to the presence of polar atoms in all molecules except benzene, the solvent makes closer contact with them as observed by the first RDF peak at a lower radius (Figure S3). The peaks at higher radii represent the second solvation shell and so on.

**Table 1:** Average Radius of the First Solvation Shell (r_max_) from MM and QM/MM Conformations of the Reduced and Oxidized States for Each Solute^a^

Molecule	Surface	*r*_max_ (Å)	
		MM conformations	QM/MM conformations
benzene	reduced	3.43	3.52
	oxidized	3.43	3.48
phenol	reduced	2.73	2.73
	oxidized	2.68	2.63
phenolate	reduced	2.73	2.73
	oxidized	2.93	2.83
indole	reduced	3.78	2.83
	oxidized	3.78	2.73
lumiflavin	reduced	3.73	2.78
	oxidized	3.93	2.78

a All atoms heavier than hydrogen were included in the RDF calculations.

As shown in Table 1, the *r*_max_ values are similar between the two oxidation states for all systems. However, the *r*_max_ values show a significant difference between the MM and QM/MM conformations for indole and lumiflavin in both oxidation states. This difference is also reflected in the RDF plot for these two molecules (Figure S3). Interestingly, the first smaller peak disappears for the MM conformations, indicating lower polarity for the heteroatoms in MM simulations. This variation is likely due to differences in the fixed partial charges in MM simulations and polarizable charges in each step of the QM/MM simulations. For example, the partial charge of the phenol oxygen atom is similar in both MM and QM/MM simulations, leading to similar RDF in QM/MM and MM conformations (Figure S4a). In contrast, the nitrogen atom of indole has a smaller negative charge in QM/MM simulations compared to the MM simulations (Figure S4b). Therefore, variation in force field parametrization process may contribute to slightly different solute–solvent interactions.

### Analysis of VEG (Δ*E*) Sampling

We have calculated VEG for 500 conformations of each system and in each oxidation state. The ensemble average VEG (AVEG) is presented in Table 2. Since the QM/MM simulations were shorter in length, we analyzed first two principle component of 500 QM/MM conformations to ensure that the conformations are uncorrelated. This analysis was performed for reduced surface of benzene, phenol, phenolate, indole, and oxidized surface of lumiflavin. Figure S5 shows that there is no correlation between the principal component 1 (PC1) vs principal component 2 (PC2), PC1 vs VEG (at cutoff 0.00), and PC2 vs VEG (at cutoff 0.00). Therefore, the QM/MM conformations are independent of each other. Then, to ensure convergence, we calculated the running average of the VEGs for both MM (Figure S6) and QM/MM sampling (Figure S7). The running averages show that 500 snapshots are sufficient to converge the VEGs for each system. Furthermore, the distribution of VEG values (Figures S8 and S9) display a single peak, confirming the convergence.

**Table 2:** VEGs (in eV) of all Systems Calculated Using ωB97MV/MM for “QM Cutoff 0.0” and “QM Cutoff 7.5” on MM and QM/MM Conformations^a^

Molecule	Surface	MM conformations		QM/MM conformations	
		QM cutoff 0.0	QM cutoff 7.5	QM cutoff 0.0	QM cutoff 7.5
benzene	reduced	9.75	9.32	9.44	9.03
	oxidized	4.91	4.95	4.72	4.76
phenol	reduced	9.23	8.80	8.89	8.45
	oxidized	4.20	4.18	3.99	3.92
phenolate	reduced	7.80	8.01	7.59	7.81
	oxidized	2.80	3.36	2.57	3.11
indole	reduced	8.12	7.91	8.16	7.79
	oxidized	4.03	4.09	3.64	3.67
lumiflavin	reduced	5.74	5.93	6.12	6.24
	oxidized	2.02	2.31	2.31	2.58

a The 6-31+G* basis set was used for calculations.

### Conformational Sampling Impacts VEGs Differently for Different Molecules and Oxidation States

To check the impact of the sampling protocol on VEGs, we calculated AVEG values from MM and QM/MM conformations with two different QM region selection schemes: QM cutoff 0.0 and QM cutoff 7.5. The differences in AVEG from the MM and QM/MM conformations at both QM cutoffs depend on the choice of redox center and the oxidation state (Table 2).

For simplicity, we will first compare the AVEG values for the QM cutoff 0.0 calculations. For benzene, phenol, phenolate, and the oxidized state of indole, AVEG from MM conformations is overestimated by ∼0.2–0.4 eV compared to AVEG from QM/MM conformations. However, for the reduced state of indole, the difference in AVEG values between the sampling protocols is negligible (0.03 eV). In contrast, lumiflavin exhibits the opposite trend, AVEG from MM conformations is underestimated by 0.4 and 0.3 eV for the reduced and oxidized conformations, respectively, compared to AVEG from QM/MM conformations.

The different trends for different molecules suggest that the choice of sampling protocol significantly influences the AVEG values in a molecule-specific manner. In a related study, the HOMO energy level of the nucleobases acquired from the MM conformations was found to be overestimated by 0.5 eV compared to those from QM/MM conformations.^23^ In contrast, Dybeck et al. reported that replacing MM potential with QM/MM potential improved the mean average deviation in solvation free energy for a set of organic molecules by only 0.3 kcal/mol (∼0.01 eV) compared to experimental data.^60^ Additionally, other studies demonstrated that both MM and QM/MM free energy perturbation methods yield identical binding free energies within statistical uncertainty.^61,62^ Therefore, the observed differences can be attributed to differences in sampling techniques, types of molecules, and the calculated observable.

We also examined the change in AVEG between the two QM/MM schemes: QM cutoff 0.0 and QM cutoff 7.5 for both sampling protocols. As shown in Table 2, the differences in AVEG between the two QM cutoffs are sensitive to the charge of the redox center. A similar difference is observed for the two QM cutoffs in both conformational sampling approaches. For positively charged systems (e.g., the oxidized state of benzene, phenol, and indole), the differences are minimal (<0.1 eV), whereas, for negatively charged conformations such as for phenolate and reduced lumiflavin, the differences are slightly higher, around 0.2 eV. However, the two QM/MM schemes show higher differences (0.3–0.6 eV) for neutral systems. Specifically, AVEG decreases for the reduced surface of benzene, phenol, and indole, and increases for the oxidized surface of phenolate and lumiflavin. The AVEG at QM cutoff 7.5 from the QM/MM conformations shows the closest agreement with experimental vertical ionization energy for closed-shell conformations, as discussed in more detail in our previous study.^63^ Experimental data is unavailable for the open-shell form of any of these molecules due to the transient nature of these species. This analysis suggests that the sampling protocol and the size of the QM region notably influence the VEGs. Therefore, to investigate the dependence of VEG further on the QM region, we extended the QM/MM VEG calculations to various QM sizes.

### AVEG Converges Faster with Respect to QM Size for Charged Conformations

Table 2 highlights the difference between the AVEG calculated with two distinct QM cutoff radii. The change in AVEGs with the inclusion of more solvent in the QM region can be attributed to a more robust treatment of the short-range solvent polarization, which is known to influence redox properties.^64^ Therefore, to further investigate how the choice of the QM region affects VEGs, we calculated the VEG for a range of different QM cutoff radii, starting from 4.0 to 7.0 Å in 0.5 Å increments. For this “cutoff analysis”, we have only used the conformations obtained from the QM/MM sampling. Thus, for a single conformation, we calculated VEG at nine different cutoff radii, including 0.0 and 7.5 Å. Figure 4 shows the plots of AVEGs with respect to the size of the QM region. In the figure, we categorize neutral species such as reduced benzene, phenol, and indole as the “neutral surface”, while positively or negatively charged species such as oxidized benzene, phenol, and indole are denoted as the “charged surface”. We observed that the AVEG converged very quickly for the charged surface, while for the neutral surface, the convergence occurred at a longer QM cutoff. Therefore, the neutral states have a stronger dependence on the size of the QM region compared to the charged species.

**Figure 4:**
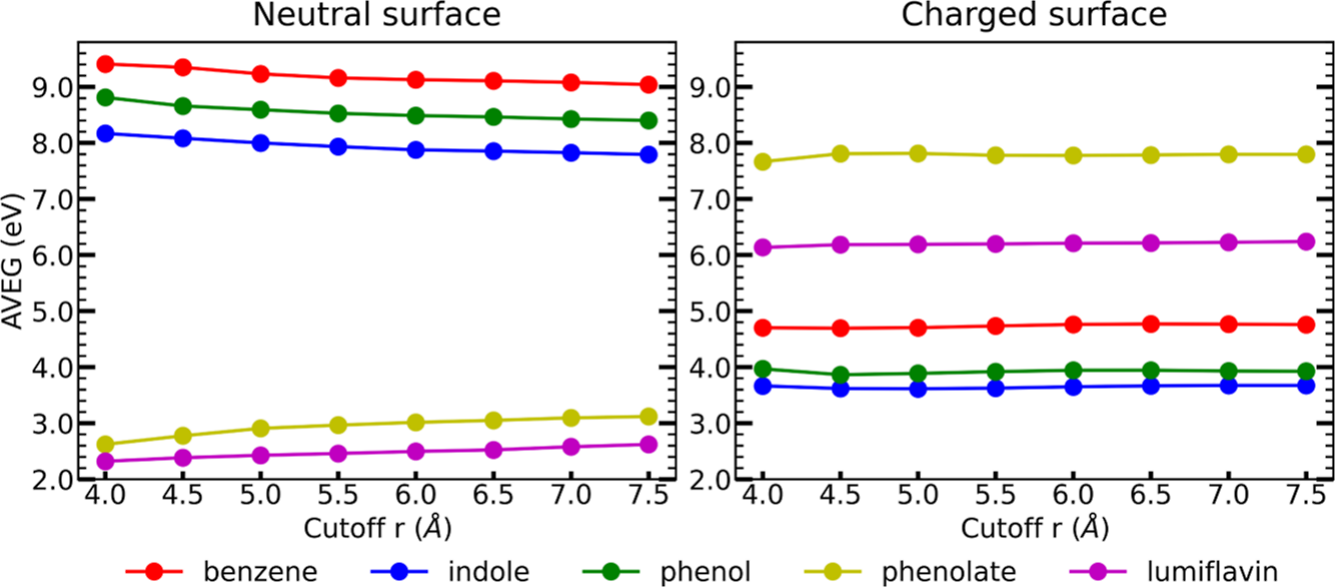
Average vertical energy gap (AVEG) with different QM cutoff (*r*) values. Here, *r* is the radius of the QM region in the single-point QM/MM energy calculations.

The differences in AVEG values for neutral surface between QM cutoff 7.0 and QM cutoff 7.5 are very minimal, indicating convergence. The highest difference in AVEG is observed in neutral lumifalvin between QM cutoff 7.0 and QM cutoff 7.5. This is attributed to the larger size of lumiflavin compared to other solute molecules. We do not expand the QM region beyond 7.5 Å to keep the computational cost manageable. Such a large QM region was shown to provide an accurate description of solvent polarization in a prior report.^7^ Furthermore, AVEG decreases with increasing QM cutoff radius for neutral benzene, phenol, and indole (all reduced), whereas for neutral phenolate and lumiflavin (both oxidized), AVEG increases as the QM cutoff radius increases. For charged species, the AVEG dependence on the QM cutoff is weaker, as indicated by the nearly horizontal fits in Figure 4 (right). This is likely due to the dominant electrostatic interactions in charged systems, which are less sensitive to changes in the QM region size.

Sterling and co-workers reported a −0.37 eV redox-shift when going from QM cutoff 0.0 to QM cutoff 5.0 for an aqueous phenol system and a shift of −0.33 eV for indole, which is strikingly similar to our observations.^7^ For a fixed QM region, Bravaya and co-workers examined solvent polarization effects by comparing QM/point charges (QM/PC) and QM/effective fragment potential (EFP).^6^ Similar to our observations, they also found that the effect of solvent polarization is dependent on the charge of the redox center. However, they found a higher dependence for AVEG with the size of the solvation shell for charged species such as oxidized state of phenol. This difference could be attributed to the fact that they examined the polarization effect based on the presence or absence of a solvent, while in our study, we varied the approach to capture the polarization effect using MM or QM/MM methods. If we compare the AVEG between QM cutoffs 0.0 and 7.5 (Table 2), we find that the difference is more pronounced in neutral conformations. Therefore, correcting for the QM treatment of solute–solvent interactions does not nullify the limitations imposed by the conformational samplings.

We have also investigated the effect of the QM region selection approach on the convergence of AVEGs. We used two selection criteria for this purpose. The first selection was based on the radius from the center of mass of the solute to the oxygen atom of water (COM cutoff). The second selection was based on the shortest distance from any solute atom to the oxygen atom of water (atom cutoff). We observed that the atom cutoff provides convergence of VEG values with fewer calculations. However, the number of water molecules included in the QM region is much larger with the atom cutoff distance, compared to the same COM cutoff value. Nevertheless, the converged VEG values used in both approaches are very similar (Table S3). We expect to observe a much smaller difference for more symmetric molecules under investigation. Since the observable remains unchanged and the COM cutoff provides a single, uniform radius that streamlines automation and cross-comparison, we have employed the COM cutoff criteria in this article.

### Neutralizing Counterions do Not Impact the AVEG Values

We added counterions to neutralize the system while performing conformational sampling of a charged solute. Since the observed trends in VEGs depend on the oxidation state of a molecule, we examined the solute-counterion interactions. First, we calculated the distance between the center of mass of the redox center and the counterion, and the results are presented in Figure S10. Our analysis indicated that the distance between the redox center and counterion changed significantly in the MM sampling, where the ion approached within 10 Å of benzene, phenol, and phenolate. For indole and lumiflavin, the population of the counterion away from the redox center in MM conformations can be attributed to the nonuniform electrostatic interactions between the molecule and the ion.

Previous studies highlighted the impact of counterion concentration on spectroscopic properties and solvation free energy.^65,66^ The variation in ion distribution between MM and QM/MM simulations aligns with findings from ref 67, which suggested that counterion’s conformations may not be adequately sampled in a 10 ps QM/MM simulation. However, they observed a strong linear correlation between the VEG of flavin and related molecules and the electrostatic potential of the surrounding protein and solvent environment. In contrast, our study found that, despite these notable positional changes in MM conformations, there does not appear to be a direct correlation between VEG and the counterion’s position.

In our study, we only used a single counterion to neutralize the system rather than maintaining an ionic strength. As shown in Table S4, we categorized 500 conformations into three groups based on the solute-counterion distance (*r*, in Å) as r < 10, 10 ≤ *r* ≤ 20 and *r* > 20, and then calculated AVEG for each category. Table S4 shows that, regardless of the distance the AVEG remained consistent. Because we did not observe a significant change in AVEGs based on the position of the single counterion, we can conclude that the variation in AVEG based on oxidation state does not arise from artifacts related to counterion placement.

### Electron Correlation Impacts QM/MM and MM Conformations Similarly

The discussion in the preceding sections has revealed that careful treatment of solvent polarization by increasing the QM region provides AVEGs comparable to experimental results. Furthermore, we use coupled-cluster singles and doubles equation-of-motion ionization potential (CCSD-EOM-IP) method^68^ to examine the effect of including electronic correlation in VEGs. Since EOM-IP is a very expensive method, we have only performed this calculation for QM cutoff 0.0. We computed VIE using the EOM-IP method for the reduced states of benzene, phenol, phenolate, and indole, on the same set of MM and QM/MM conformations. Table S5 shows the comparison between AVEG calculated using DFT and EOM-IP methods. We observe that regardless of the sampling protocol, the EOM-IP values show an improvement in AVEG by 0.1–0.3 eV compared to the DFT results. In all cases, the EOM-IP VEG values from QM/MM conformations provide closer agreement with experimental VIE than those from MM conformations. The computed EOM-IP VIE of phenol and phenolate from QM/MM conformations are 8.65 and 7.31 eV, respectively. Thus, the EOM-IP for phenol falls within the broad range of experimental spectra, while the EOM-IP for phenolate is overestimated by 0.21 eV.^14^ For QM/MM conformations of benzene, EOM-IP shifts VEG by 0.12 eV, compared to DFT values, and provides good agreement with the experimental value for benzene-1-water cluster.^69^ However, in spite of 0.2 eV improvement from both MM and QM/MM conformations, EOM-IP values of indole are 0.5 eV overestimated compared to the experimental data.^70^

Overall, the results from Table S5 show a consistent improvement in VEGs due to the correlation effects. More importantly, these effects are similar in both the MM and QM/MM conformations, meaning that the improvement is uniform across different conformations and can be corrected by a constant shift.

### Free Energy Change and Redox Potential

Since the larger QM region in the QM/MM single-point calculations offers the best agreement, we calculated the free energy change for oxidation using AVEG from QM cutoff 7.5 in the LRA approach. The free energy change for the oxidation process using eq 2, is presented in Table 3. A comparison of the data in Table 3 shows that the differences in Δ*G*°_ox_ between MM and QM/MM conformational sampling range from approximately 0.2 to 0.4 eV. For benzene, phenol, phenolate, and indole, the Δ $G_{ox}^{{}^{\circ}}$ values from MM conformations are higher than those from QM/MM conformations. In contrast, the Δ $G_{ox}^{{}^{\circ}}$ for lumiflavin is about 0.3 eV lower for MM conformations compared to QM/MM conformations.

**Table 3:** Free Energy Change (Δ $G_{ox}^{{}^{\circ}}$ , in eV), and One-Electron Oxidation Potential (E° vs SHE, in V) Calculated Using VEG Data From DFT/MM Calculations at QM Cutoff 7.5 on MM and QM/MM Conformations and Experimental Oxidation Potential (Expt. E° vs SHE, in V)

Molecule	MM conformations		QM/MM conformations		Expt. *E*°
	Δ $G_{ox}^{{}^{\circ}}$	*E*°	Δ $G_{ox}^{{}^{\circ}}$	*E*°	
benzene	7.14	2.70	6.90	2.46	2.72^a^ ^,^ ^71^
phenol	6.49	2.05	6.12	1.68	1.0–1.5 ^72–74^
phenolate	5.67	1.25	5.46	1.02	0.83–0.86 ^75–77^
indole	6.00	1.56	5.73	1.29	1.24 ^78^
lumiflavin	4.12	–0.32	4.41	–0.03	0.101^b^ ^,^ ^79^

a : In acetonitrile.

b : For FMN.

Overall, similar to AVEG, the Δ $G_{ox}^{{}^{\circ}}$ values from QM/MM samplings show better agreement with the previously reported values, compared to those from MM samplings. For instance, the calculated Δ $G_{ox}^{{}^{\circ}}$ for phenol and phenolate from QM/MM conformations are in close agreement with ref 6. The Δ $G_{ox}^{{}^{\circ}}$ of indole is 5.52 eV, calculated from the data in ref 7, which corresponds well with our observations. Thus, the Δ*G*°_ox_ of the redox process can vary significantly depending on the conformational sampling methods used. A similar trend is also observed in nonequilibrium sampling techniques. For instance, Δ $G_{ox}^{{}^{\circ}}$ values for Fe ${\left(H_{2}O\right)}_{6}^{2+/3+}$ and Ru ${\left(H_{2}O\right)}_{6}^{2+/3+}$ complexes are 5.82 and 5.14 eV, respectively, calculated by QM/MM thermodynamic integration.^80^ In contrast, the corresponding Δ $G_{ox}^{{}^{\circ}}$ values from MM simulations are 3.5 ^81^ and 10.34 ^82^ eV. Therefore, the difference in free energy of oxidation between MM and QM/MM conformation sampling is heavily influenced by the system under investigation.

Finally, using eq 3, we determined the oxidation potential from the calculated Δ $G_{ox}^{{}^{\circ}}$ values. We report the values with respect to a standard hydrogen electrode (SHE) with a reference value of 4.44 V^83^ for Δ $G_{\mathrm{SHE}}^{{}^{\circ}}$ . The results in Table 3 show positive *E*° values for benzene, phenol, phenolate, and indole, indicating that these species are easier to oxidize. In contrast, lumiflavin exhibits a negative *E*° value, suggesting it is difficult to oxidize. Overall, the *E*° values derived from QM/MM and MM conformations differ by approximately 0.2–0.4 V, depending on the system. Interestingly, with the exception of benzene, *E*° values from QM/MM conformations are more aligned with the experimentally reported values (Table 3). A comparison between computed and experimental redox potentials is illustrated in Figure 5.

**Figure 5:**
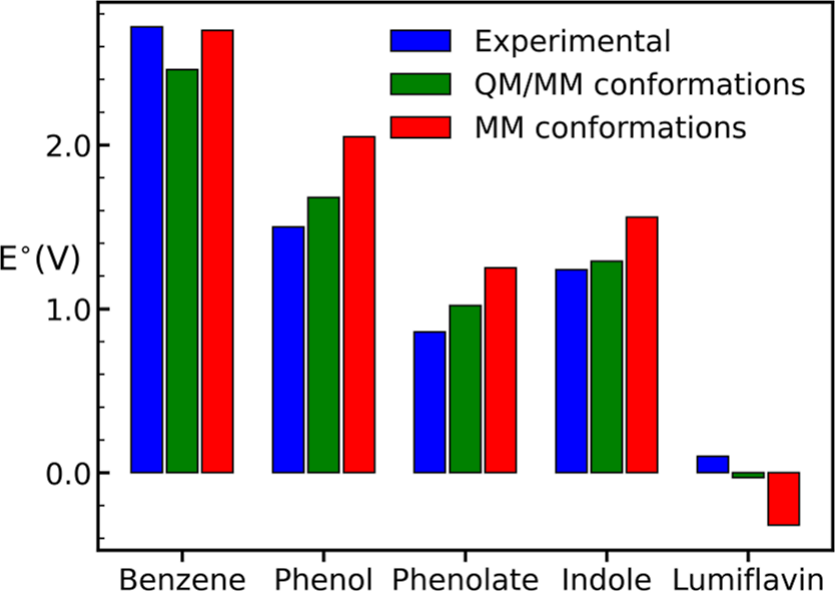
Experimental and computed (at QM cutoff 7.5) oxidation potential values (*E*°) for all the systems.

The redox potential of benzene in water is not readily available as a result of experimental difficulties. However, our computed *E*° is in good agreement with the experimental value obtained in acetonitrile (Table 3). For phenol, reported redox potential spans a wide range of *E*° values (1.0–1.5 V).^72–74^ Overall, the computed redox potentials of phenol and phenolate are slightly overestimated compared to the experimentally reported values. In contrast, the computed values for indole and lumiflavin are very close to the experimental *E*° values, although the sign is still opposite for lumiflavin. Nevertheless, the oxidation potential calculated from the QM/MM conformations agrees more with the experiential values compared to the MM conformations.

## Conclusions

In this study, we employed both MM and hybrid QM/MM dynamics simulations in an aqueous environment to estimate VEG, Δ $G_{ox}^{{}^{\circ}}$ , and *E*° for the one-electron oxidation process of various biologically relevant redox-active molecules. First, we compared the conformational sampling protocols by analyzing the structural differences in solvent arrangement around the solute. Analysis of the radial distribution function showed that except for indole and lumiflavin, the MM and QM/MM methods describe solvent rearrangement similarly. Second, we investigated how different conformational sampling methods influence the vertical energy gap and observed a 0.2–0.4 eV difference between the sampling protocols. The Δ $G_{ox}^{{}^{\circ}}$ values acquired from the MM and QM/MM conformations showed a similar difference of 0.2–0.4 eV. Finally, we compared the *E*° obtained from the MM and QM/MM conformations and also with experimental data, observing the same trend as Δ $G_{ox}^{{}^{\circ}}$ .

We examined the solvent polarization effect in VEGs with different QM sizes and obtained a consistent improvement in the VEGs, regardless of the sampling protocol used. Among the different QM region sizes, a cutoff of 7.5 Å from QM/MM conformations yielded the most accurate VEG compared to experimental data. The VEG values converge differently for different oxidation states of the molecule. Our findings reveal that the MM sampling, while more cost-effective, contains higher error when used for redox potential calculations. However, the higher error accumulated from MM sampling may be mitigated by a constant correction factor, especially for the same molecule. This will warrant careful system-specific benchmarking before estimating redox properties from MM conformational sampling. Beyond electrostatic embedding, for properties that will require incorporating MM polarization based on the QM charges, one will also need to consider the use of more sophisticated polarizable embedding approaches.^16,84–86^

## Supplementary Material



## Data Availability

Models used in this study, including PSF, PDB, and force field parameters are available at 10.5281/zenodo.14854325

## Notes

Additional details of simulations, QM/MM single-point calculations, VEG convergence, the effect of correlation, and ion distributions are provided in the Supporting Information (PDF)
