# Supplementary material for: Effects of Conformational Sampling on Computing Redox Properties Using Linear Response Approach
Source: J Phys Chem B. 2025 Jul 14;129(29):7465–74. doi: 10.1021/acs.jpcb.5c01121 (PMC12302069; doi:10.1021/acs.jpcb.5c01121)
Supplement: Supplementary file 1 [file jp5c01121_si_001.pdf]

Supporting Information: Effects of Conformational Sampling on Computing Redox Properties Using Linear Response Approach

Suman Maity,† Ronit Sarangi,† and Atanu Acharya*,†,‡

†*Department of Chemistry, Syracuse University, Syracuse, NY 13244, United States*

‡*BioInspired Syracuse, Syracuse University, Syracuse, NY 13244, United States*

E-mail: achary01@syr.edu

Contents

1 Oxidation Process **S-3**

2 Workflow **S-4**

3 Basis Set Effect on VEG **S-4**

4 QM Region Selection **S-6**

5 Radial Distribution Function **S-8**

6 System Details **S-9**

7 Principle Component Analysis (PCA) **S-10**

8 Running Average **S-11**

8.1 From MM Conformations S-11

8.2 From QM/MM Conformations S-12

9 VEG (ΔE) Distributions **S-13**

9.1 From MM Conformations S-13

9.2 From QM/MM Conformations S-14

10 Counterion Position **S-15**

1 Oxidation Process

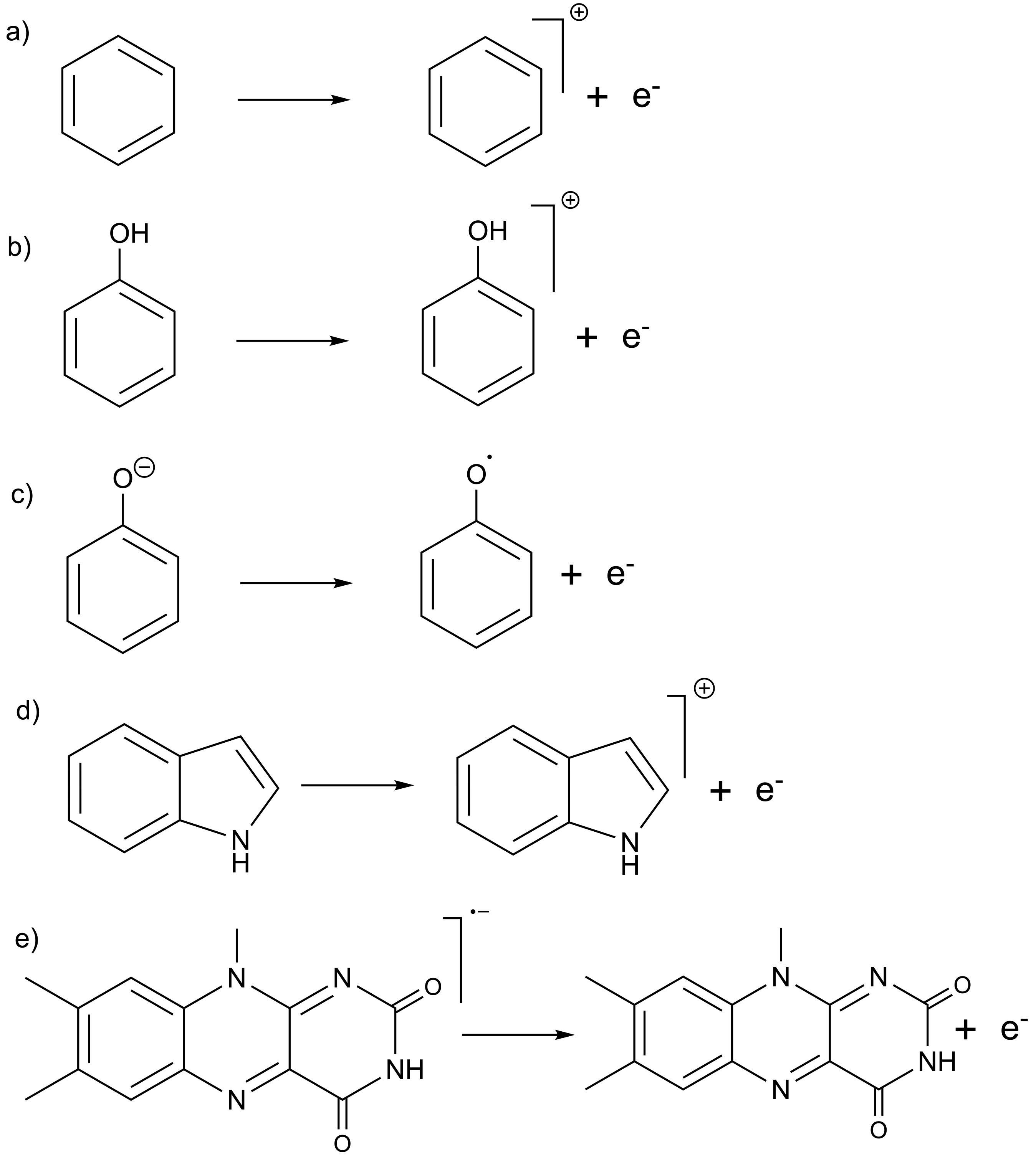

Figure S1: One electron oxidation process for a) benzene, b) phenol, c) phenolate, d) indole, and e) lumiflavin.

2 Workflow

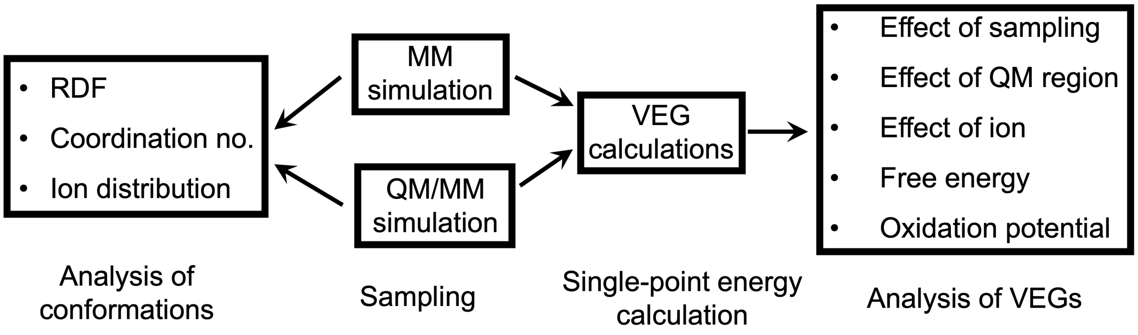

Figure S2: Basic workflow adopted in this article

3 Basis Set Effect on VEG

We have studied the basis set dependence for VEGs on the MM conformations for reduced surface of benzene, phenol, phenolate, indole and the oxidized surface of lumiflavin at QM cutoff 0.0. We used 6-31+G*, 6-311++G**, aug-cc-pVDZ, and aug-cc-pVTZ basis sets in these calculations. The AVEGs are shown in Table S1, which demonstrates that the effect of increasing basis set size on VEGs from 6-31+G* is negligible (< 0.05 eV).

Table S1: Effect of basis set size on AVEGs (in eV) on MM conformations for reduced surface of benzene, phenol, phenolate, indole, and oxidized surface of lumiflavin. All calculations were performed using the ωB97MV functional with QM cutoff 0.0 using a DFT/MM scheme.

Basis set	AVEG (eV)				
	Benzene	Phenol	Phenolate	Indole	Lumiflavin
6-31+G*	9.75±0.34	9.23±0.34	7.80±0.35	8.12±0.33	2.02±0.31
6-311++G**	9.80±0.34	9.27±0.35	7.86±0.35	8.18±0.33	2.07±0.31
aug-cc-pVDZ	9.73±0.34	9.20±0.35	7.80±0.35	8.13±0.33	2.01±0.31
aug-cc-pVTZ	9.73±0.34	9.20±0.35	7.81±0.35	8.13±0.33	2.03±0.31

Table S2: Average number of waters in the QM region for different QM cutoffs for single point QM/MM calculations.

Cutoff (Å)	Surface	Number of waters[a]				
		Benzene	Phenol	Phenolate	Indole	Lumiflavin
4.0	Reduced	2.57 ± 1.03	2.72 ± 1.10	3.49 ± 1.07	2.62 ± 0.99	2.31 ± 1.10
	Oxidized	2.38 ± 1.18	2.59 ± 1.14	1.76 ± 1.04	2.39 ± 1.04	2.45 ± 0.94
4.5	Reduced	5.51 ± 1.77	6.99 ± 1.32	8.12 ± 1.55	5.67 ± 1.33	5.91 ± 1.42
	Oxidized	6.83 ± 1.80	7.12 ± 1.55	5.76 ± 1.63	5.86 ± 1.35	5.50 ± 1.38
5.0	Reduced	11.59 ± 2.59	12.26 ± 2.03	13.51 ± 2.01	10.19 ±1.65	9.17 ± 1.51
	Oxidized	14.23 ± 1.91	13.2 ± 2.01	12.05 ± 2.07	10.72 ± 1.70	8.95 ± 1.48
5.5	Reduced	18.72 ± 2.61	18.72 ± 2.10	19.8 ± 2.24	16.70 ± 2.01	13.27 ± 1.72
	Oxidized	20.83 ± 1.99	19.97 ± 1.95	19.13 ± 2.27	17.75 ± 2.09	13.50 ± 1.76
6.0	Reduced	25.71 ± 2.24	25.67 ± 2.33	26.47 ± 2.14	25.13 ± 2.22	19.37 ± 2.10
	Oxidized	26.88 ± 1.91	26.35 ± 2.02	25.93 ± 2.53	25.53 ± 2.32	19.90 ± 2.32
6.5	Reduced	33.44 ± 2.28	33.04 ± 2.13	34.45 ±2.62	33.09 ± 2.26	28.30 ± 2.56
	Oxidized	33.58 ± 2.13	33.74 ± 2.10	33.20 ± 2.48	33.61 ± 2.37	28.71 ± 2.58
7.0	Reduced	42.09 ± 2.74	42.0 ± 2.52	44.19 ± 2.67	42.08 ± 2.58	37.88 ± 2.47
	Oxidized	42.67 ± 2.32	43.20 ± 2.46	42.24 ± 2.68	42.99 ± 2.70	38.45 ± 2.74
7.5	Reduced	52.8 ± 3.02	53.3 ± 2.89	55.5 ± 2.80	52.5 ± 2.76	47.8 ± 2.70
	Oxidized	54.2 ± 2.81	54.4 ± 2.63	53.1 ± 3.04	54.0 ± 2.76	48.8 ± 2.60

[a]: The average number of waters in the QM region and its standard deviation is calculated across 500 snapshots for each surface.

4 QM Region Selection

The average number of waters in the QM region in the VEG calculations is presented in Table S2, which shows a gradual increase with increasing cutoff values. Here, cutoff values for the QM region are selected as the distance between the center of mass of the solute and the oxygen atom of the water (Approach 1: COM cutoff). We investigated the effect of the QM region selection approach on the convergence of AVEGs on the oxidized surface of lumiflavin for QM/MM conformations. Secondly, we selected the QM region based on the shortest distance between the solute and the oxygen atom of the water solvent (Approach 2: atom cutoff), and then we varied the QM region from 2.0 Å to 5.0 Å with a 0.5 Å increment. Table S3 shows that the AVEG converges faster in Approach 2 compared to Approach 1. This is probably due to the presence of more water molecules in the QM region for the same cutoff value. Thus, the change in AVEG does not depend on how we select the QM region, but rather on the number of water molecules in the QM region.

Table S3: Effect of QM region selection approach in single point QM/MM calculations on convergence of VEG tested on oxidized surface of lumiflavin on QM/MM conformations.

Approach 1	Cutoff selection: distance between the center of mass of the solute and the oxygen atom of water							
	4.0	4.5	5.0	5.5	6.0	6.5	7.0	7.5
nwat	2.45±0.94	5.50±1.38	8.95±1.48	13.50±1.76	19.90±2.32	28.71±2.58	38.45±2.74	48.8±2.60
AVEG (eV)	2.31±0.34	2.38±0.34	2.43±0.35	2.46±0.34	2.49±0.35	2.52±0.34	2.58±0.34	2.62±0.34
Approach 2	Cutoff selection: the shortest distance between the oxygen of water and the solute							
	2.0	2.5	3.0	3.5	4.0	4.5	5.0	-
nwat	0.9±0.31	2.27±1.09	15.59±2.45	29.16±2.84	40.0±2.97	51.28±2.97	63.96±3.03	-
AVEG (eV)	2.26±0.34	2.26±0.34	2.48±0.35	2.59±0.34	2.60±0.35	2.62±0.34	2.63±0.34	-

5 Radial Distribution Function

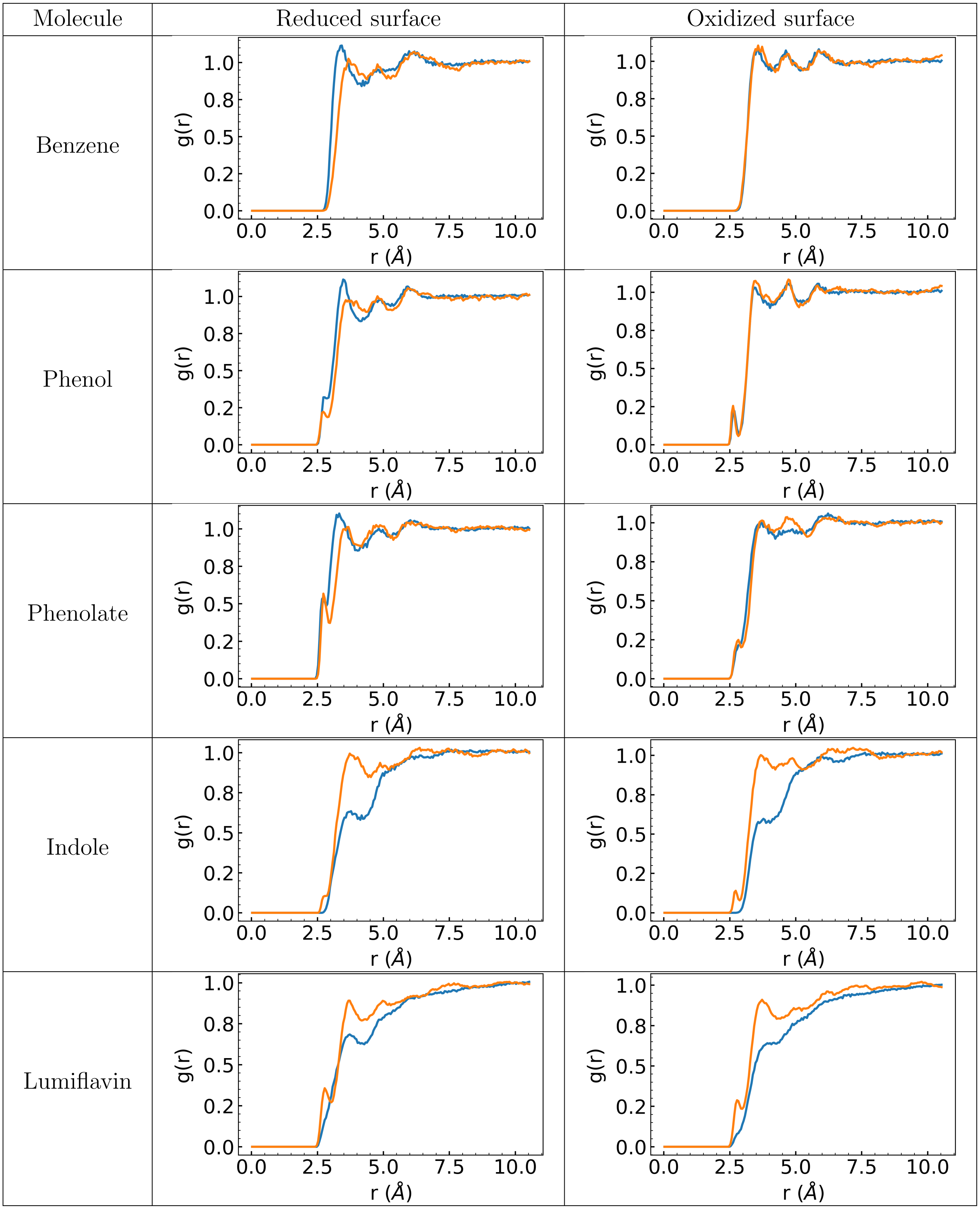

Figure S3: Radial distribution function (RDF) from MM (blue) and QM/MM (orange) conformations, calculated between heavy atoms of the solute and the oxygen atom of water.

6 System Details

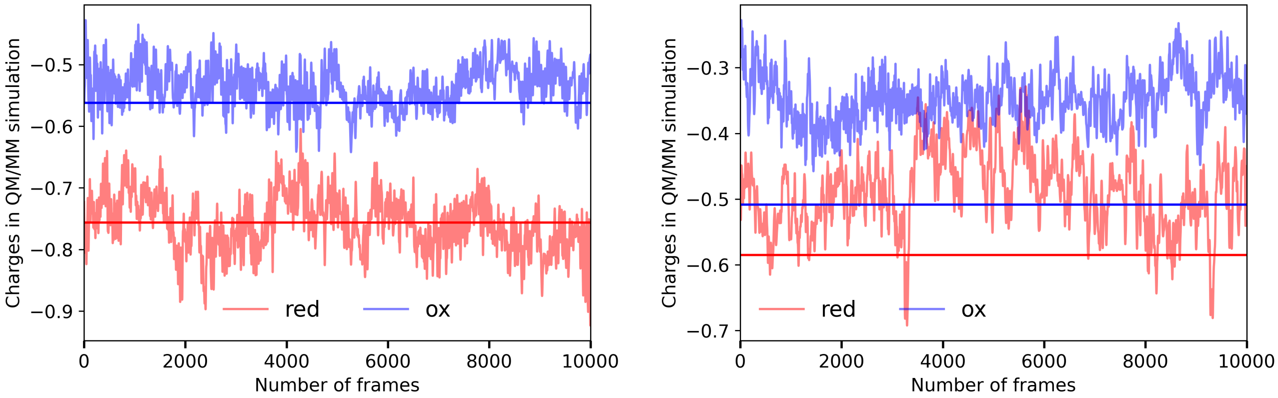

(a) Phenol **(b) Indole**

Figure S4: Partial charges of (a) oxygen atom of phenol and (b) nitrogen atom of indole, during QM/MM simulations, and the straight lines represent the fixed charge in MM simulations.

7 Principle Component Analysis (PCA)

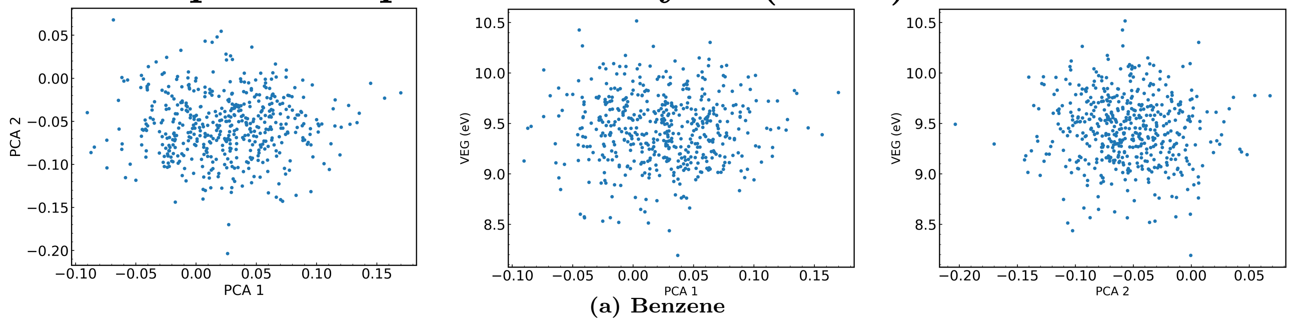

(a) Benzene

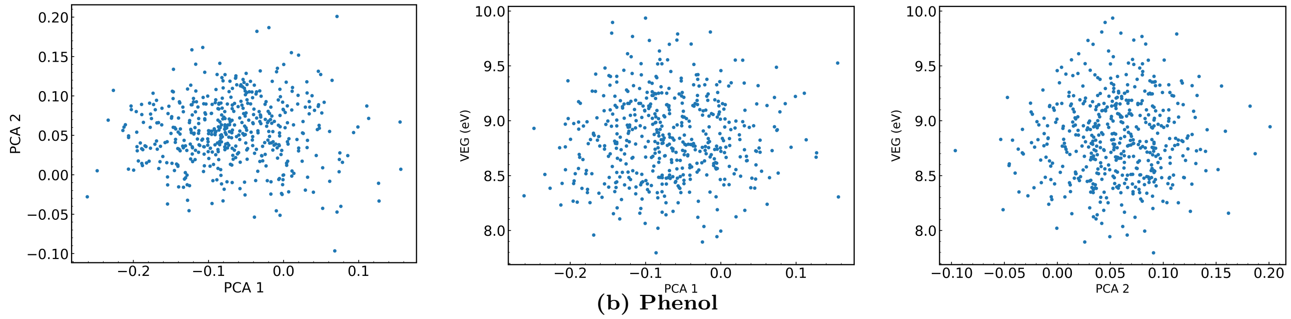

(b) Phenol

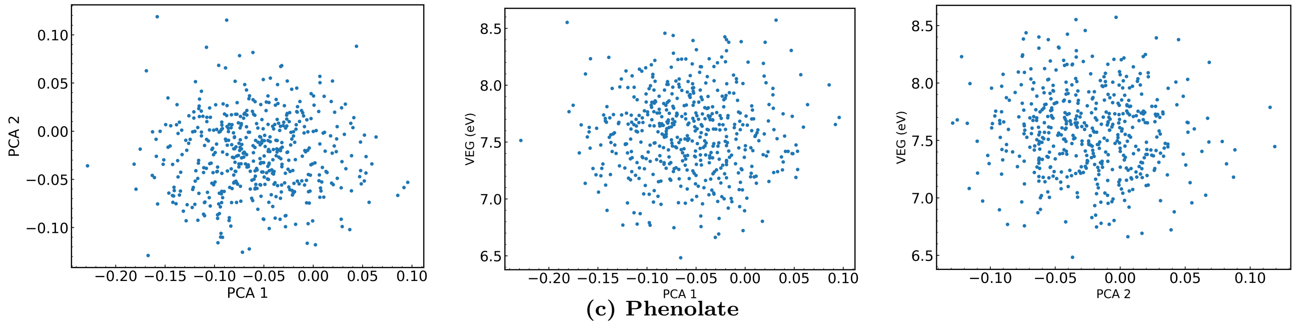

(c) Phenolate

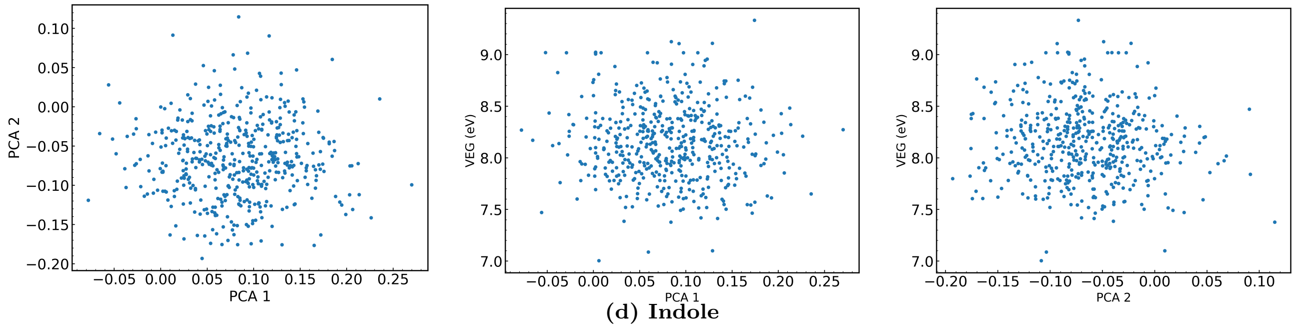

(d) Indole

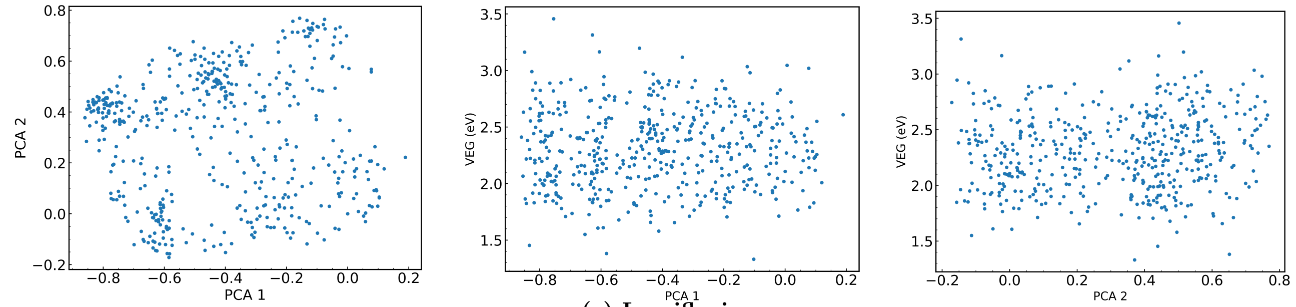

(e) Lumiflavin

Figure S5: Principle component analysis (PCA) of concatenated QM/MM conformations for reduced surface of benzene, phenol, phenolate, indole, and oxidized surface of lumiflavin. For each system, we plotted PC1 vs PC2, PC1 vs VEG, and PC2 vs VEG. In each case, the scattered nature of the plot confirms that QM/MM conformations are uncorrelated.

8 Running Average

8.1 From MM Conformations

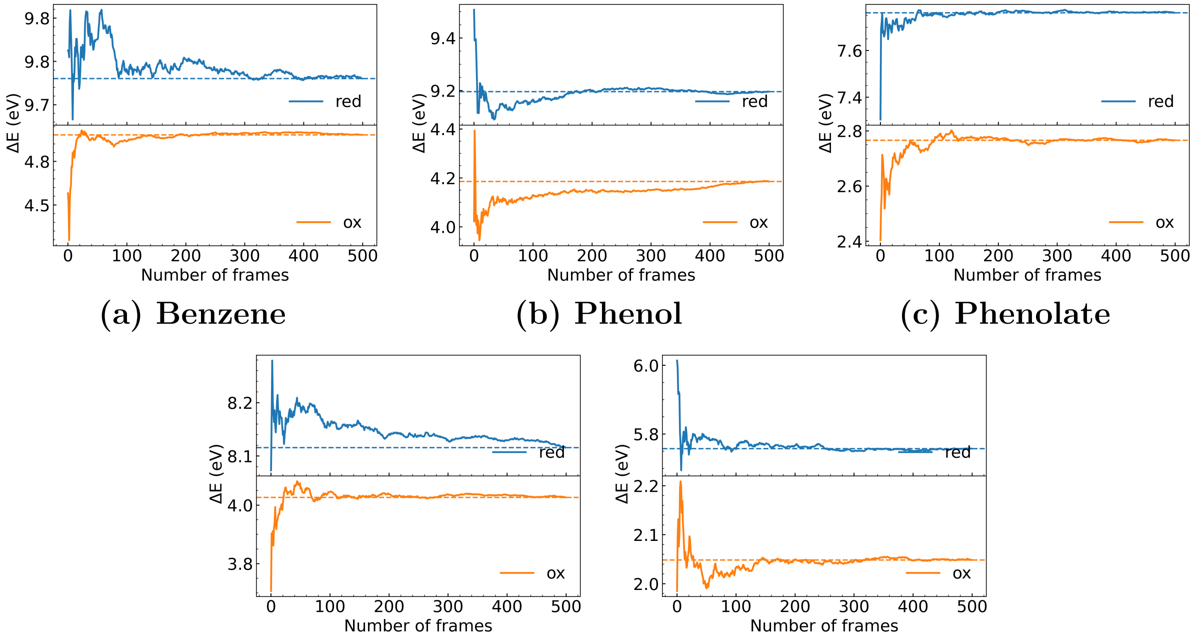

(a) Benzene (b) Phenol (c) Phenolate

(d) Indole (e) Lumiflavin

Figure S6: Running average of VEG at the reduced (top) and oxidized (bottom) surfaces from QM/MM single-point calculations with MM conformations.

8.2 From QM/MM Conformations

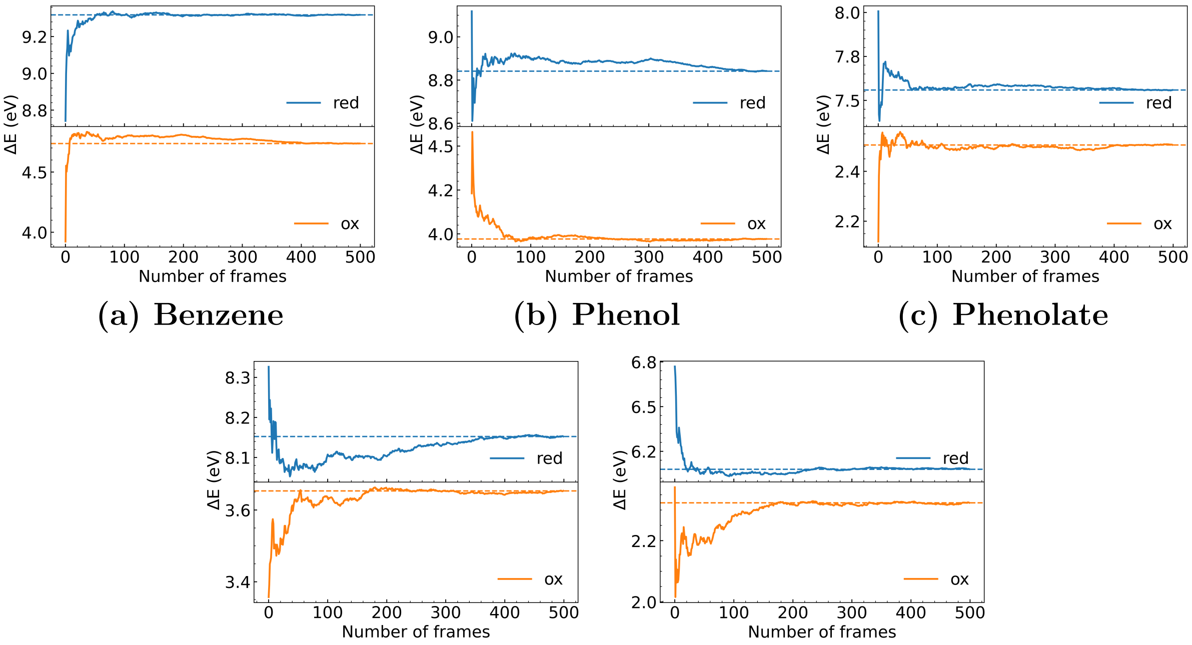

(a) Benzene (b) Phenol (c) Phenolate

(d) Indole (e) Lumiflavin

Figure S7: Running average of VEG at the reduced (top) and oxidized (bottom) surfaces from QM/MM single-point calculations with QM/MM conformations.

9 VEG (ΔE) Distributions

9.1 From MM Conformations

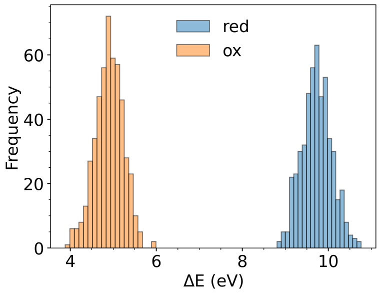

(a) Benzene

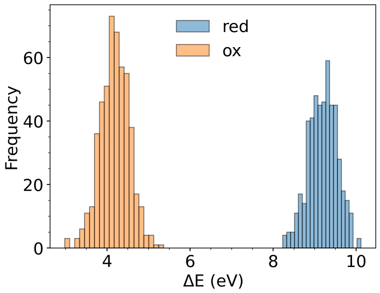

(b) Phenol

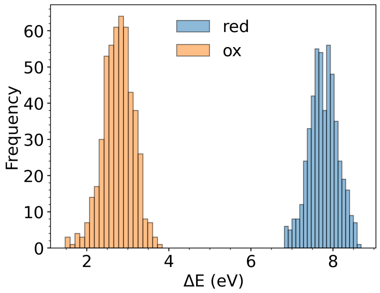

(c) Phenolate

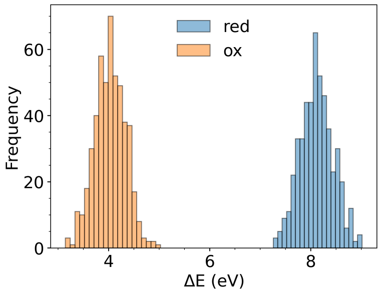

(d) Indole

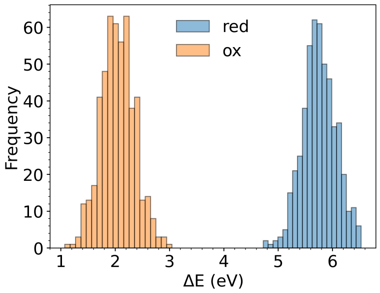

(e) Lumiflavin

Figure S8: VEG distributions from "QM cutoff 0.0" at the reduced and oxidized surfaces from DFT/MM calculations on MM conformations.

9.2 From QM/MM Conformations

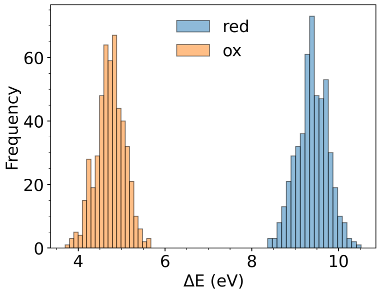

(a) Benzene

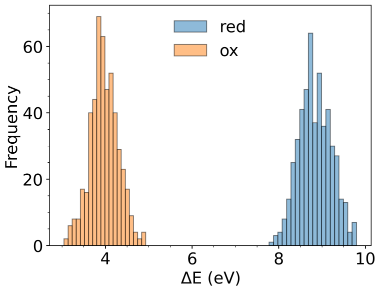

(b) Phenol

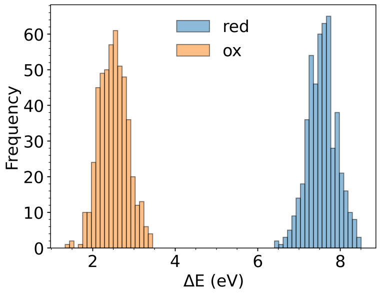

(c) Phenolate

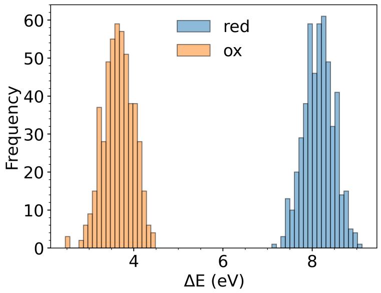

(d) Indole

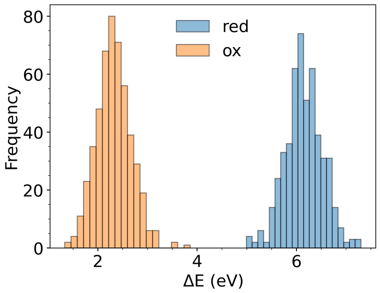

(e) Lumiflavin

Figure S9: VEG distributions from "QM cutoff 0.0" at the reduced and oxidized surfaces from QM/MM single-point calculations with QM/MM conformations.

10 Counterion Position

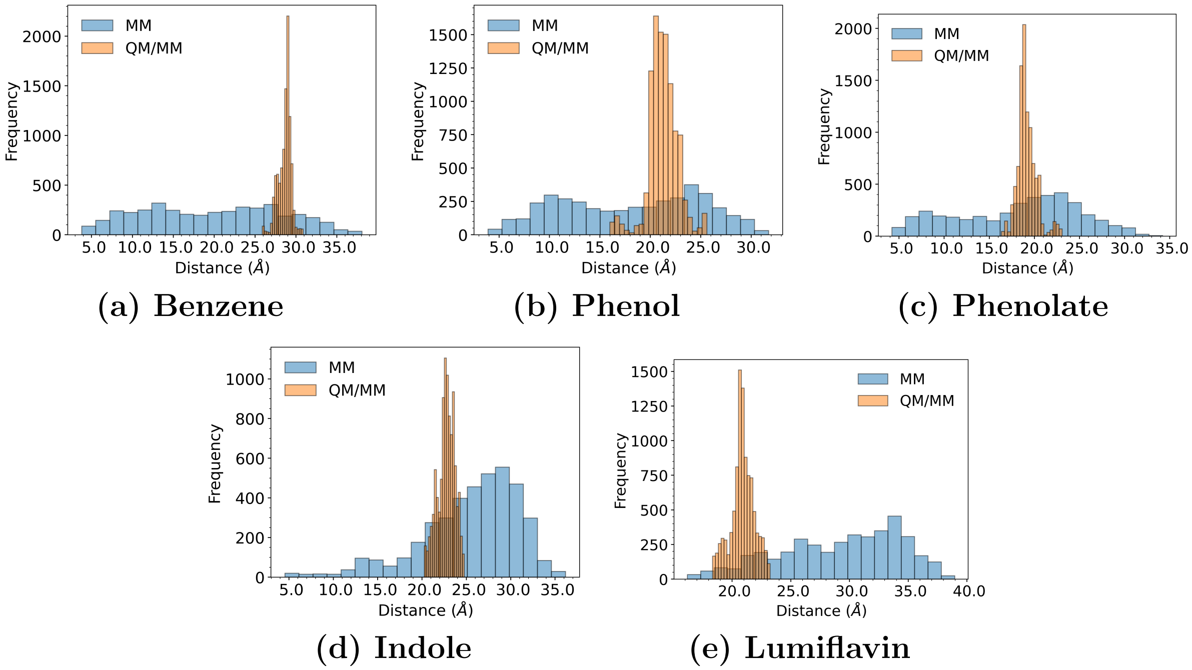

Figure S10: Distribution of distances between the center of mass of the redox center and the ion from MM (blue) and QM/MM (orange) conformations.

Table S4: Dependence of VEGs on the position of the counterion from QM cutoff 0.0 calculations with MM conformations.

Molecule	Distance (Å)	Number of snapshots	AVEG (eV)
Benzene	$r < 10$	79	4.90 ± 0.35
	$10 \leq r \leq 20$	171	4.92 ± 0.34
	$r > 20$	250	4.89 ± 0.32
Phenol	$r < 10$	78	4.20 ± 0.36
	$10 \leq r \leq 20$	198	4.23 ± 0.37
	$r > 20$	224	4.18 ± 0.34
Phenolate	$r < 10$	82	7.79 ± 0.35
	$10 \leq r \leq 20$	173	7.83 ± 0.35
	$r > 20$	245	7.77 ± 0.35
Indole	$r < 10$	7	3.73 ± 0.27
	$10 \leq r \leq 20$	69	4.01 ± 0.25
	$r > 20$	424	4.04 ± 0.31
Lumiflavin	$r < 10$	0	-
	$10 \leq r \leq 20$	26	5.80 ± 0.28
	$r > 20$	474	5.73 ± 0.31

Table S5: VEGs (in eV) from DFT, VIEs (in eV) from EOM-IP, and differences between DFT and EOM-IP (E_{shift}) values for a subset of systems

Molecule	MM conformations			QM/MM conformations		
	DFT	EOM	E_{shift}	DFT	EOM	E_{shift}
Benzene	9.75 ± 0.34	9.60 ± 0.34	0.15	9.40 ± 0.36	9.28 ± 0.34	0.12
Phenol	9.23 ± 0.34	9.04 ± 0.35	0.19	8.86 ± 0.38	8.65 ± 0.37	0.21
Phenolate	7.80 ± 0.35	7.51 ± 0.35	0.29	7.58 ± 0.35	7.31 ± 0.35	0.27
Indole	8.12 ± 0.33	7.92 ± 0.32	0.20	8.14 ± 0.34	7.91 ± 0.36	0.23
